# Osmophobia in adult and juvenile headache patients

**DOI:** 10.1186/1129-2377-16-S1-A22

**Published:** 2015-09-28

**Authors:** Giorgio Zanchin

**Affiliations:** Interdepartmental Centre for Headache and Drug Abuse, Padua University School of Medicine, Padua, Italy

## Introduction

Osmophobia (Os), defined as a perception of odors as unbearable during the headache attacks, has been described since ancient times as an accompanying symptom of migraine.

More recently authors demonstrated that Os is much more relevant in migraine patients (M), with (MA) or without aura (MO), than in various primary headaches (PH). It has also been put in evidence that Os is very specific in the differential diagnosis between M and tension-type headache (TTH). Consequently, the 2004 edition of ICHD included in the Appendix (A1.1) Os among the proposed alternative diagnostic criteria of MO. In January 2013, a provisional revised version (ICHD-3 beta) of the previously released classification was offered for the approval of the scientific community. In this version Os disappeared from the Appendix, without any explanation.

## Aim and methods

To understand this choice, we reviewed and analyzed the available scientific data on Os features in M, and more specifically the usefulness of Os in the differential diagnosis of various types of primary headaches. An open search was performed on MedLine, which yelded 50 articles listing among their keywords “Osmophobia”. We considered the papers issued after the release of ICHD-2, finding 42 articles which appeared between 2005 and 2015, of which 19 had been published since 2013. Among these, 29 were eligible: while 18 papers investigated Os only as an accompanying symptom in M and/or various PH, 11 also focused on its relevance for differential diagnosis. We calculated the cumulative values of sensibility and specificity of Os in the differential diagnosis of M vs TTH.

## Results and conclusions

Literature reports a much higher prevalence of Os in M than in various PH, particularly TTH[1]. Even if sensibility and specificity range from 25% to 86% and 69% to 100% respectively in different papers, all published data support the usefulness of Os in the differential diagnosis between M and TTH. Calculated cumulative values demonstrate a high specificity, between 87% and 98%, of Os in the diagnosis of M in adulthood. As far as Os in children is concerned, it appears to have an even more important role since the presence of Os in a child presenting as suffering of TTH results to be a prognostic marker of the future clinical development of MO[2].

In conclusion, published data consistently support the inclusion of Os among the M diagnostic criteria. On this ground of robust evidence, the unexplained decision to remove Os from the diagnostic criteria of M in ICHD-3 beta appears methodologically unjustified. A revision of this choice is therefore strongly recommended.
